# Bridging functional and anatomical neural connectivity through cluster synchronization

**DOI:** 10.1038/s41598-023-49746-2

**Published:** 2023-12-17

**Authors:** Valentina Baruzzi, Matteo Lodi, Francesco Sorrentino, Marco Storace

**Affiliations:** 1 DITEN, University of Genoa, Via Opera Pia 11a, 16145 Genova, Italy; 2 Mechanical Engineering Department, University of New Mexico, Albuquerque, NM 87131 USA

**Keywords:** Applied mathematics, Applied physics

## Abstract

The dynamics of the brain results from the complex interplay of several neural populations and is affected by both the individual dynamics of these areas and their connection structure. Hence, a fundamental challenge is to derive models of the brain that reproduce both structural and functional features measured experimentally. Our work combines neuroimaging data, such as dMRI, which provides information on the structure of the anatomical connectomes, and fMRI, which detects patterns of approximate synchronous activity between brain areas. We employ cluster synchronization as a tool to integrate the imaging data of a subject into a coherent model, which reconciles structural and dynamic information. By using data-driven and model-based approaches, we refine the structural connectivity matrix in agreement with experimentally observed clusters of brain areas that display coherent activity. The proposed approach leverages the assumption of homogeneous brain areas; we show the robustness of this approach when heterogeneity between the brain areas is introduced in the form of noise, parameter mismatches, and connection delays. As a proof of concept, we apply this approach to MRI data of a healthy adult at resting state.

## Introduction

The complex spatio-temporal patterns of activity exhibited by the brain derive from the interplay between the neural dynamics of cortical areas and their connectivity. From a modeling standpoint, one of the most challenging problems is the inference of network parameters in a brain model to reproduce empirically observed activity, namely the so-called inverse problem^1^. One of the pillars for deriving reliable models that under specific conditions can reproduce experimentally observed human brain functions is the growing availability of neuroimaging techniques, which allow the detection of patterns of activity across neural units that encode objects, concepts, or states of information^2^. Various techniques based on imaging data have been developed to extract functional and structural connectivity matrices of the human/animal cortex, but the way brain function is shaped by the underlying anatomical substrate is far from being understood^3^. Moreover, despite its widespread use to probe the structural connectivity of the brain in both clinical and basic neuroscience, dMRI fiber tractography produces anatomical connectomes subject to quite a large degree of uncertainty, due to well-known pitfalls of this technique in terms of anatomical accuracy^4^. A second research path that is attracting a growing interest is how to employ network theory and nonlinear dynamics to build brain models based on imaging data^5–12^.

The orchestrated activity of neural populations has been postulated to be one of the key mechanisms underlying brain functions^13^. At a microscopic level, individual neurons exhibit hardly predictable firing rates, with a non-negligible stochastic component^14^. At a macroscopic level, however, we see the emergence of regular oscillations of the local field potential (LFP) in specific brain areas, including one or more populations, such as in the case of the cortical rhythms in the cerebral cortex^15^. We do not observe complete (or identical) synchronization between brain areas, i.e., a situation in which all variables of all areas converge to the same dynamical state; synchronization is usually only approximate, with brain areas behaving coherently, and transient, with synchronized communities reoccurring intermittently in time and across scanning sessions^16^. One of the most common ways to non-invasively detect the presence of coherence among brain areas is to analyze the correlation between the blood oxygen level-dependent (BOLD) signals measured in these areas through functional magnetic resonance imaging (fMRI).

It is commonly agreed that brain functions strongly depend on anatomy, other than neural dynamics. Various efforts have been made to determine if functional connectivity and structural connectivity can be predicted from each other^17^: data-driven statistical models^18–20^ that do not assume any specific mode of interaction among neuronal populations; models emerging from network science and telecommunication engineering^21^ that conceptualize functional interactions as the superposition of elementary signaling events on the underlying anatomical network; biophysical dynamical models^22–24^ that describe the collective dynamics of a neuronal ensemble in terms of their mean firing rate, which capture both local fluctuations and influence from other connected regions, and exhibit a rich repertoire of oscillations, synchrony, and waves. Many studies have investigated whether and how resting-state functional connectivity can be inferred from experimentally measured structural connectivity^22,24,25^, and have found that strength, persistence, and spatial statistics of functional connections are constrained by the large-scale anatomical structure of the human cerebral cortex. Some studies have also proposed analytical approaches to derive linear relationships between functional and structural connectivity matrices, based on neural field theory^26^ and spectral graph theory^27^.

In this paper, we investigate an approach to reconcile information about structural connectivity and information on synchronous clusters obtained from fMRI data. Our proposed method provides a biophysical dynamical model in terms of a network, composed of weighted links and nodes representing brain areas. The network links (i.e., its topology) and weights are derived from an optimized version of the original structural connectivity matrix, obtained by imposing the network emergent dynamics to be compatible with the synchronous clusters evidenced by fMRI scans. The results are robust with respect to heterogeneity in the parameters of both network and nodes, the presence of noise, and connection delays. The main novelty element of this paper is the connection we establish between anatomical and functional data based on the study of cluster synchronization at different granularities. To the best of our knowledge, the concepts of cluster synchrony and equitable partitions have not been previously leveraged to relate the functional and structural connectomes of the brain.

As a proof of concept, we apply the method to public data obtained from 10 fMRI and 10 dMRI scans executed across a month of one healthy adult at resting state. Results obtained for two additional subjects of the same public dataset are provided in “Supplemental Material”, Note 2, and are aligned with the results presented here.

## Results

### Proposed method

The proposed method leverages structural and functional data in the form of connectivity matrices. In particular, structural connectivity matrices aggregate the information on fiber density between the different areas considered as network nodes; this information is derived from fiber tractography reconstruction techniques, which allow assessing the presence of neural tracts from dMRI data. Functional connectivity matrices aggregate the information on the correlation between the BOLD signals measured in the different areas through fMRI.

**Figure 1:**
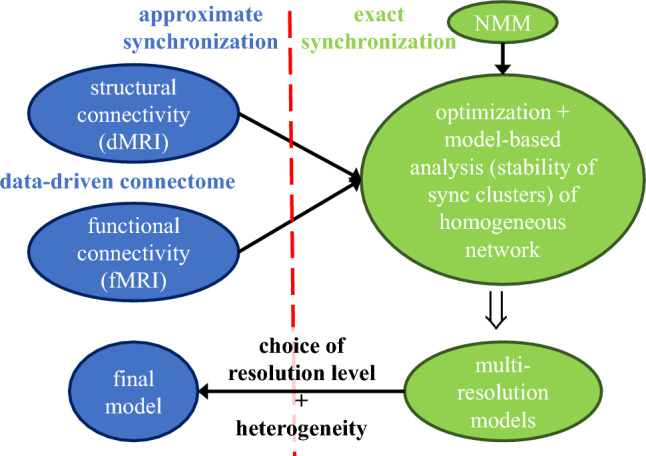
Summary of the proposed method. We start (top-left ovals) from data-driven structural (functional) connectomes, derived from dMRI (fMRI) scans. Each functional connectome evidences the correlation between the neural activity of different brain areas, and then the presence of approximately synchronized clusters, formed by areas with high correlation. There is a degree of uncertainty on the number of clusters the areas can be best aggregated into. Each structural connectome reflects the brain anatomy, with some uncertainty in the weight of each connection. Right (green) ovals: model-based method used to bridge functional and structural connectivity. The reference (homogeneous) network model has an initial topology defined by the structural connectome and node dynamics imposed by a NMM. The uncertainty on the clusters is managed through a multi-resolution approach, by analyzing different cluster partitions. For each resolution level, the uncertainty on the connection weights is reduced by optimizing them to enforce the existence of the cluster synchronous solution of that level. Bottom-left oval: one resolution level is chosen, and the corresponding model is made heterogeneous, thus obtaining again approximate synchronization.

Nonlinear dynamics provides tools for studying complete cluster synchronization^28–30^; when applying these tools to experimental data, for which synchronization is only approximate, a convenient first-order assumption is to deal with homogeneous nodes. On the one hand, this could be unrealistic, because in a real neuronal network each area is expected to be unique, i.e., from a modeling standpoint, to have its own parameter values, as the corresponding neuronal populations are heterogeneous with respect to multiple physical properties. On the other hand, the identification of these heterogeneities (and corresponding model parameters) from measured data is highly nontrivial and depends on the quality and quantity of data, the noise inherent to measurements of biological systems, and the complexity of the chosen model.

Figure 1 illustrates the main elements of the proposed method, which starts from functional and structural connectomes derived from fMRI and dMRI scans, respectively. The functional data evidences the presence of approximate synchronization (namely, high correlation) between the neural activity of specific brain areas. The areas with high correlation are assumed to be part of the same *synchronous cluster*. In order to reduce the uncertainty on the weights of the structural connectome^4^ we look for network models, with different granularity/resolution in terms of clusters, that are compatible with the clusters evidenced by the functional connectivity analysis. The key elements of the method are the following:

- We use the network *topology* defined by the structural connectome as a starting point.
- We use functional data to identify *clusters* of nodes with coherent activity through a hierarchical clustering algorithm, with multiple resolution levels.
- We add *dynamics* to the nodes through a neural mass model (NMM)^31^, choosing the network model to be *homogeneous* in the nodes, and focus the analysis on exact cluster synchronization; at this stage, we neglect higher-order model factors that pertain to the node features.
- We resort to the concept of equitable clusters^32,33^ in order to optimize the weights of the structural connectome with the aim of making the network compatible with the *existence* of clusters of highly functionally-correlated areas; this is done by changing the weights as little as possible from their experimentally measured values and by taking into account their measured uncertainty.
- We find a suitable range for a scale factor of the weights by applying the master stability function (MSF) approach^34^ in order to ensure the *stability* of the compatible synchronous clusters.

After the steps above are taken, one can choose the model with the most suitable resolution level, according to the specific objective, and check the extent of the model robustness by reintroducing heterogeneity in either the connection weights and delays or the nodes. This leads to a set of perturbed models compatible with the data, in which both functional and anatomical connectivity are incorporated. We emphasize that the chosen unperturbed model is able to reproduce the correlation in the BOLD signals observed experimentally in fMRI data (i.e., the approximate synchronization between brain areas) with connection weights that differ from the dMRI connectivity matrix by an amount that is comparable with the uncertainty introduced by the measuring process.

#### Network model

We consider a weighted and undirected network graph with *N* nodes, each representing a cortical area. Edges represent long-range connections between the cortical areas. This graph can be described by a symmetric structural connectivity matrix $A_0$, with entries $a_{0_{ij}}$ that are the connection weights. For a given subject, $A_0$ is built by normalizing and averaging the available $M_d$ structural connectomes, which are measured in time periods that are short enough that anatomical connectivity at this scale does not vary significantly^24,35^. Information on the uncertainty of each weight is stored in a matrix $\Sigma _{A_0}$, where each entry is the variance of the weight among the $M_d$ normalized connectomes. A delay $\tau _{ij}$ is assigned to the connection between nodes *i* and *j*, according to the distance between the nodes and the propagation speed. By assigning dynamics to each node, the network of *N* coupled neural oscillators is described by the following general set of equations ($i,j=1,\ldots ,N$):

1 $$\begin{aligned} \dot{\pmb {x}}_{i}(t) = \pmb {F}(\pmb {x}_{i}(t))+ \pmb {\Gamma }\Bigg (\pmb {x}_{i}(t), \sigma \sum _l \sum _j a_{ij}^l\pmb {G}(\pmb {x}_j(t-\tau ^l))\Bigg ) \end{aligned}$$

where $\pmb {x}_{i}\in \mathbb {R}^m$ are the state variables of node $i=1,\dots ,N$, $\pmb {F}:\mathbb {R}^m\rightarrow \mathbb {R}^m$ describes the dynamics of an isolated node, $\pmb {\Gamma }:\mathbb {R}^m \times \mathbb {R}^m\rightarrow \mathbb {R}^m$ describes the coupling between nodes, and $a_{ij}$ is the strength of the coupling from node *j* to node *i*. The parameter $\sigma$ controls the overall strength of the connections and $\pmb {G}:\mathbb {R}^m\rightarrow \mathbb {R}^m$ is the coupling function. The delays $\tau _{ij}$ are quantized over *L* values $\tau ^l$, with $l=1,\ldots ,L$. This quantization is necessary for computational purposes, and fully heterogeneous delays will be reintroduced *a posteriori*.

#### Identification of clusters of nodes with coherent activity

**Figure 2:**
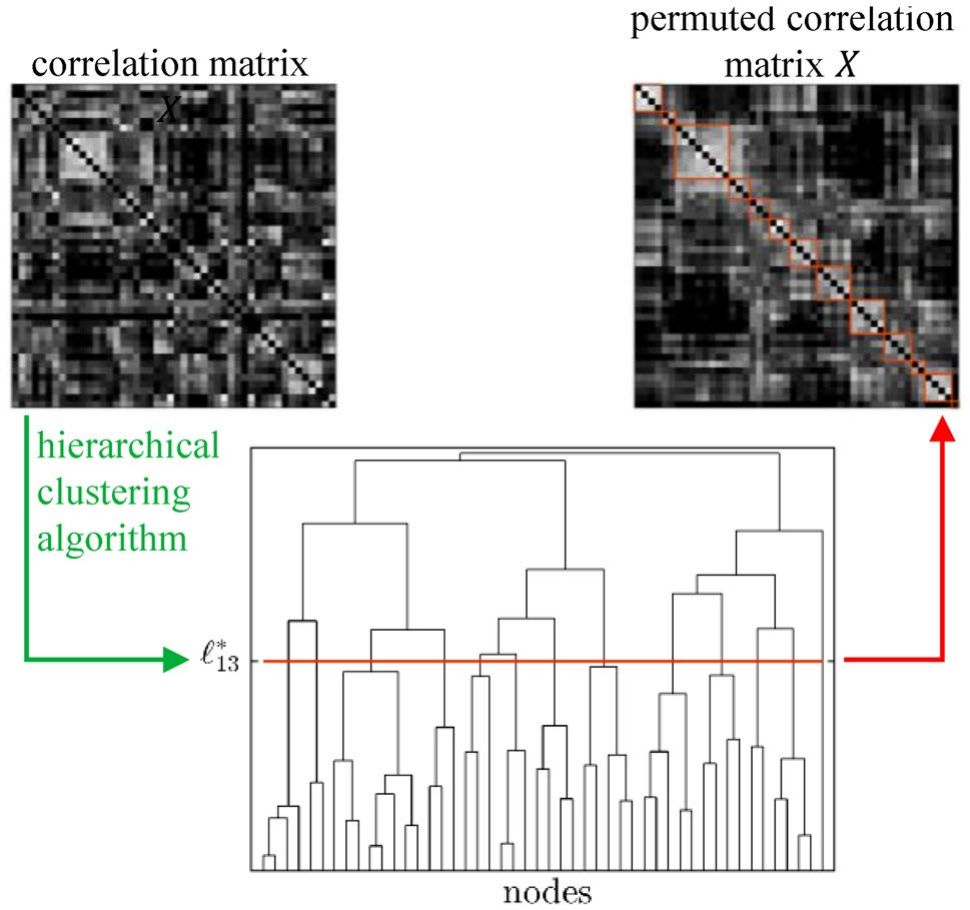
Given a correlation matrix, it is possible to identify the clusters of nodes that exhibit coherent activity with a hierarchical clustering approach. The obtained hierarchical clustering can be visualized in the form of a dendrogram, where nodes appear on the horizontal axis according to a permutation that places nodes belonging to the same cluster adjacent to one another. Depending on the level $\ell$ at which the dendrogram is cut horizontally, the corresponding clustering is described by the sub-trees originating from each branch cut by the line: for example, the red line cuts the dendrogram at the level corresponding to 13 clusters. The correlation matrix can be re-arranged based on the obtained permutation, so that the clusters can be evidenced along the diagonal.

The functional connectomes refer to the same *N* brain areas and are in the form of correlation matrices, in which each entry describes the pair-wise temporal correlation between the activity of two brain areas. Given $M_f$ functional connectomes measured from the same subject in the same conditions, it is possible to identify the clusters of nodes that exhibit coherent activity through a hierarchical clustering approach, as commonly done in the literature^36–39^. Hierarchical clustering is applied to each functional connectome, and the resulting partitioning can be visualized as a dendrogram, an example of which is shown in Fig. 2 (see Methods for details).

**Figure 3:**
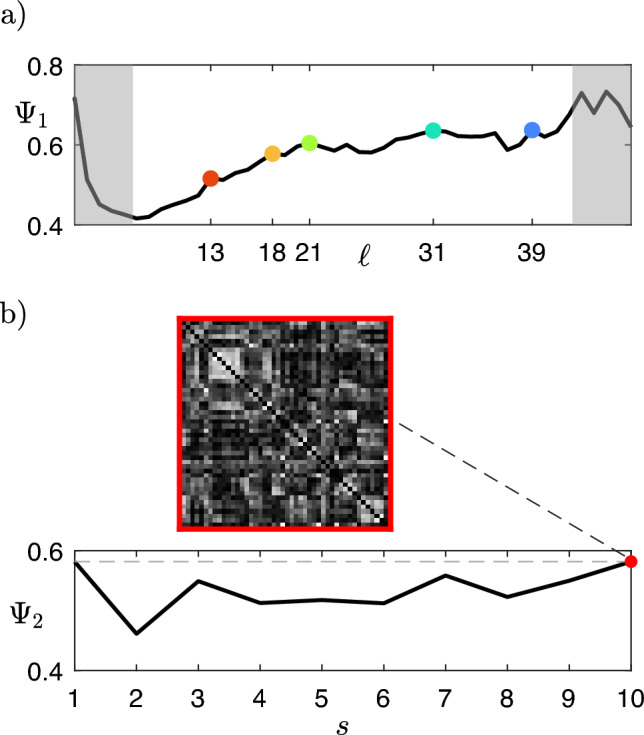
(**a**) Cumulative (across the 10 fMRI sessions) similarity index $\Psi _1$, based on the Fowlkes and Mallows comparison measure, for each dendrogram level $\ell$. $\Psi _1$ measures the consistency of the corresponding clustering across the fMRI sessions. Specific levels $\ell ^*_k$ (corresponding to *k* clusters) are selected as local maxima of this curve (colored dots, same color code as in Figs. 5 and 6). We chose to discard local maxima that are not in ascending order and trivial partitions with too few or too many clusters (grey areas, corresponding to those in panel a) of Fig. 5). (**b**) Cumulative (across levels $\ell ^*_k$) similarity index $\Psi _2$ for each of the 10 sessions. $\Psi _2$ indicates how much the clustering obtained from a session is similar to the clusterings from all other sessions. The fMRI session corresponding to the maximum value of $\Psi _2$ (red dot, corresponding to the correlation matrix $X^*$ shown above) is chosen as the most representative of the subject.

A certain variability across the dendrograms obtained from different fMRI sessions is expected. We aim to identify the set of node partitions that best describes the functional connectivity of the subject at different scales (i.e., at different levels $\ell$ of the dendrogram); to this end, we define the similarity index $\Psi _1(\ell )$, which measures the consistency of clusterings at different levels $\ell$ across the $M_f$ fMRI sessions. The local maxima of $\Psi _1(\ell )$ allow selecting $M_\ell$ levels $\ell ^*_k$ (each one cutting *k* branches of the dendrogram, thus identifying *k* clusters), which are collected in a set $\mathcal {L}^*=\{\ell ^*_k\}$ (see Fig. 3a). The reference fMRI session $s^*$ is identified through the similarity index $\Psi _2(s)$, which provides an indication of how much the clustering obtained from a session is similar to the clusterings from all other sessions; $s^*$ is chosen as the maximum value of $\Psi _2(s)$ and the selected correlation matrix is referred to as $X^*$ (see Fig. 3b). The similarity indices $\Psi _1(\ell )$ and $\Psi _2(s)$ are calculated by leveraging the Fowlkes and Mallows method^40^, which allows comparing two partitions by calculating the comparison measure $0\le B\le 1$, as explained in detail in Methods.

To check if the structural connectivity matrix $A_0$ is already compatible with the clusters emerging from the analysis of functional data, for each level $\ell ^*_k$ we simulate the network dynamics for an array of values of $\sigma$ (see Eq. (1)) and different initial conditions; we then compute an average Fowlkes and Mallows matching index $\bar{B}$ by comparing each partition obtained in simulation with the target partition associated with $X^*$. This procedure is detailed in Methods. If $A_0$ turns out to be inadequate to reproduce the clustering activity of the network (i.e., $\bar{B}\le B_{th}$, with $B_{th}$ user-defined threshold), we take the next steps of the method. We heuristically set $B_{th} = 0.7$.

#### Refining the structural connectivity matrix according to synchronized clusters

As a first approximation, we model the identified clusters of nodes with coherent activity as synchronized clusters. This model must admit these synchronized clusters as a stable synchronous solution. To impose this constraint, there are two distinct issues to be addressed: one is ensuring the existence of the desired cluster-synchronous solution, and the other one is ensuring its stability. The existence of a cluster-synchronous solution requires that, for proper initial conditions, nodes in the same cluster synchronize. This solution is also stable if, under small enough perturbations, the system state goes back to the same synchronous clusters.

We leverage here a fundamental principle: the existence of a particular set of self-sustained synchronized clusters in a network graph with homogeneous nodes can be ruled out or not based on the network topology, independently from the dynamical model of the nodes; in particular, the existence of an equitable partition for a given network is a necessary condition for the existence of a cluster synchronous solution^41,42^. We remark that at this point we are only considering the existence of a cluster synchronous solution, not its stability; the possibility of convergence on such a solution depends also on the node dynamics, i.e., the functions $\pmb {F}$ and $\pmb {\Gamma }$ in Eq. (1)^43^.

Let us first consider the case with homogeneous delays, i.e. $L=1$. A partition (and the corresponding coloring) is *equitable* if all nodes with color *p* receive the same overall input from the nodes of color *q*, for $p, q=1,...,k$. For each level $\ell _k^*$, given the set of target clusters, we want to obtain a structural connectivity matrix $A_k$ for which they constitute an equitable partition.

The structural connectivity matrix $A_0$ is derived from diffusion MRI data, and, as such, it has positive entries, is symmetrical, and has null diagonal entries. The optimized matrix $A_k$ is also constrained to (i) have positive entries, (ii) be symmetric, and (iii) have null diagonal entries, to maintain the original features of the structural connectivity matrix. From the variance matrix $\Sigma _{A_0}$, a ‘reliability’ matrix $\hat{\Sigma }_{A_0}$ is defined as $\hat{\Sigma }_{A_0}=\max (\Sigma _{A_0})-\Sigma _{A_0}+\epsilon$, so that the entries with low variance have high reliability and vice versa; $\epsilon$ is an arbitrarily small quantity with the only function of avoiding zero entries. $\hat{\Sigma }_{A_0}$ is included in the cost function so that the more reliable an entry is, the less likely it is to be changed significantly in the optimization process to find $A_k$. The optimization process is as follows: the columns of $A_k$ are stacked in the vector *x*; the columns of the matrix obtained multiplying entry by entry $A_0$ by $\hat{\Sigma }_{A_0}$ are stacked in the vector $\alpha$; *H* is defined as the $N^2$-size diagonal matrix with $\hat{\Sigma }_{A_0}$ entries on its diagonal; $M_1 x=0$ codifies the equitable partition conditions and $M_2 x=0$ codifies constraints (ii) and (iii). The optimal vector *x* (i.e., the matrix $A_k$) is found by solving the following optimization problem, with quadratic objective function and linear constraints, of equality and inequality:

2 $$\begin{aligned} \min _{x} \frac{1}{2}x^THx-\alpha ^Tx \end{aligned}$$

s.t.

3 $$\begin{aligned} \begin{array}{c} x>0\\ M_1 x=0\\ M_2 x=0. \end{array} \end{aligned}$$

This problem can be easily recast as a quadratic programming (QP) problem and numerically solved.

To carry out the optimization when delays $\tau _{ij}$ are quantized over multiple values (i.e. $L>1$) the structural connectivity matrix $A_0$ is split into *L* matrices $A_0^l$, each corresponding to a coupling with delay $\tau ^l$. In particular, entries of $A_0^l$ corresponding to different delays are set to 0 and $\sum _l A_0^l=A_0$. Each matrix $A_0^l$ is optimized individually, thus obtaining matrices $A_k^l$, according to the definition of equitable partitions for graphs with *L* different kinds of couplings^30^. The final optimized structural connectivity matrix for level *k* is obtained as $\sum _l A_k^l=A_k$. For high values of *L*, we would obtain extremely sparse matrices $A_0^l$, thus reducing the degrees of freedom for the optimization algorithm, since we constrain zero entries to be kept at zero.

#### Stability analysis through master stability function

The stability of the synchronous target clusters for each level $\ell _k^*$ is assessed by using the MSF approach^34^. To illustrate this step of the proposed method, we refer to a network of *N* coupled Wilson-Cowan NMMs^44^ (see Methods), which can be described by the general set of equations (1) with $m=2$ state variables:

$$\begin{aligned}{}&\pmb {x}_i=\begin{bmatrix}E_i\\ I_i\end{bmatrix},\\&\pmb {F}(\pmb {x}_i)=\begin{bmatrix}\frac{1}{\tau _E}(-E_i)\\ \frac{1}{\tau _I}(-I_i+\frac{1}{1+e^{-c(w_{EI}E_i-\theta )}})\end{bmatrix},\\&\pmb {\Gamma }(\pmb {x}_i,\sigma \sum _j a_{ij} \pmb {G}(\pmb {x}_j))=\begin{bmatrix}\frac{1}{\tau _E}\frac{1}{1+e^{-c(w_{EE}E_i-w_{IE}I_i+P+E^{syn}_i-\theta )}}\\ 0\end{bmatrix},\\&\pmb {G}(\pmb {x}_j)=\begin{bmatrix}E_j\\ 0\end{bmatrix}. \end{aligned}$$

We can denote the cluster synchronization state as $\pmb {x}_i(t)=\pmb {s}_p(t)$, where node *i* belongs to cluster $\mathcal {C}_p$. Small perturbations $\pmb {w}_{i}(t) = \pmb {x}_{i}(t)-\pmb {s}_p(t)$, stacked in the state perturbation vector ${\pmb {W}(t)}$, are introduced to investigate the stability of the synchronous state and their linearized variational equations are derived (see Methods for details).

We resort to a coordinate transformation based on simultaneous block diagonalization (SBD)^45–48^, performed through the canonical transformation matrix $$T=\left( \begin{array}{c} T_{\parallel } \\ T_{\bot } \\ \end{array} \right)$$^49^. An alternative to this method is the one proposed in^50^. The $k \times N$ submatrix $T_\parallel$ is associated with the directions *along* the synchronization manifold and the corresponding perturbations do not influence the stability of the synchronized clusters. The $(N-k) \times N$ submatrix $T_\perp$ is associated with the directions *transverse* to the synchronization manifold and the evolution of the variational equation along these directions determines the stability of the synchronized clusters.

Transverse perturbations are denoted as $\pmb {\eta }_\bot (t)=(T_\bot \otimes I_m)\pmb {W}(t)$ (where $\otimes$ is the Kronecker product and $I_m$ is a $m \times m$ identity matrix) and their variational equation is:

4 $$\begin{aligned} \begin{array}{c} \dot{\pmb \eta }_\bot (t)= \rho _1(\left\{ \pmb {s}_p(t) \right\} ) \pmb \eta _\bot (t)+ \displaystyle {\rho _2^l(\left\{ \pmb {s}_p(t) \right\} )}\pmb {\eta }_\bot (t-\tau ^l) \end{array} \end{aligned}$$

where the set $\left\{ \pmb {s}_p(\cdot ) \right\}$ collects all the synchronous solutions corresponding to the *k* clusters and $\rho _1$ and $\rho _2^l$ are time-varying matrices defined in Methods. The Lyapunov exponents for each $\pmb {\eta }_\bot$ component are calculated and the $\sigma$ intervals where the cluster synchronous solution is stable are determined as those where the maximum Lyapunov exponent (MLE) is negative.

#### Introducing heterogeneity in the network model

The assumption of exact cluster synchronization is restrictive and unrealistic when referring to experimental data from recorded brain activity. For this reason, we check if the obtained stability intervals of $\sigma$ values hold also for perturbed versions of $A_k$ that produce approximate synchronization. For small perturbation of the matrix $A_k$, this was studied in^42^. We repeat the compatibility check as described in the simulation procedure in Methods on the optimized structural connectivity matrices $A_k$ perturbed by additive Gaussian white noise with mean 0 and standard deviation $\sigma _A$. This analysis tests for up to which level of noise on the connection weights the optimized structural connectivity matrix remains compatible with the target clusters, derived from the functional connectivity matrix, in the considered $\sigma$ intervals.

Similar analyses can be carried out by adding a white Gaussian noise term $\eta _i(t)$ with standard deviation $\sigma _{\eta }$ to every node’s input or by introducing heterogeneous node parameters.

### Proof of concept of the method

In this section, the method steps are illustrated through a case study. All functional and structural connectomes used in this study are part of the Neurodata MRI Cloud database^51^. They are produced with NeuroData’s MRI Graphs pipeline^52^ from the HNU1 dataset^53^, which includes $M_f=10$ fMRI and $M_d=10$ dMRI scans of 30 healthy adults at resting state collected over the span of one month. The illustrative example presented in this paper refers to subject 0025452, which showed the highest similarity index $\Psi _1$ averaged across levels.

First, we present the results for different clustering granularities for the case with no connection delays (i.e. $\tau _{ij}=0\quad \forall \quad i,j$). Next, we analyze the case with delays $\tau _{ij}$ quantized over $L=3$ values for an intermediate granularity level $\ell ^*_k$.

#### Network model

**Figure 4:**
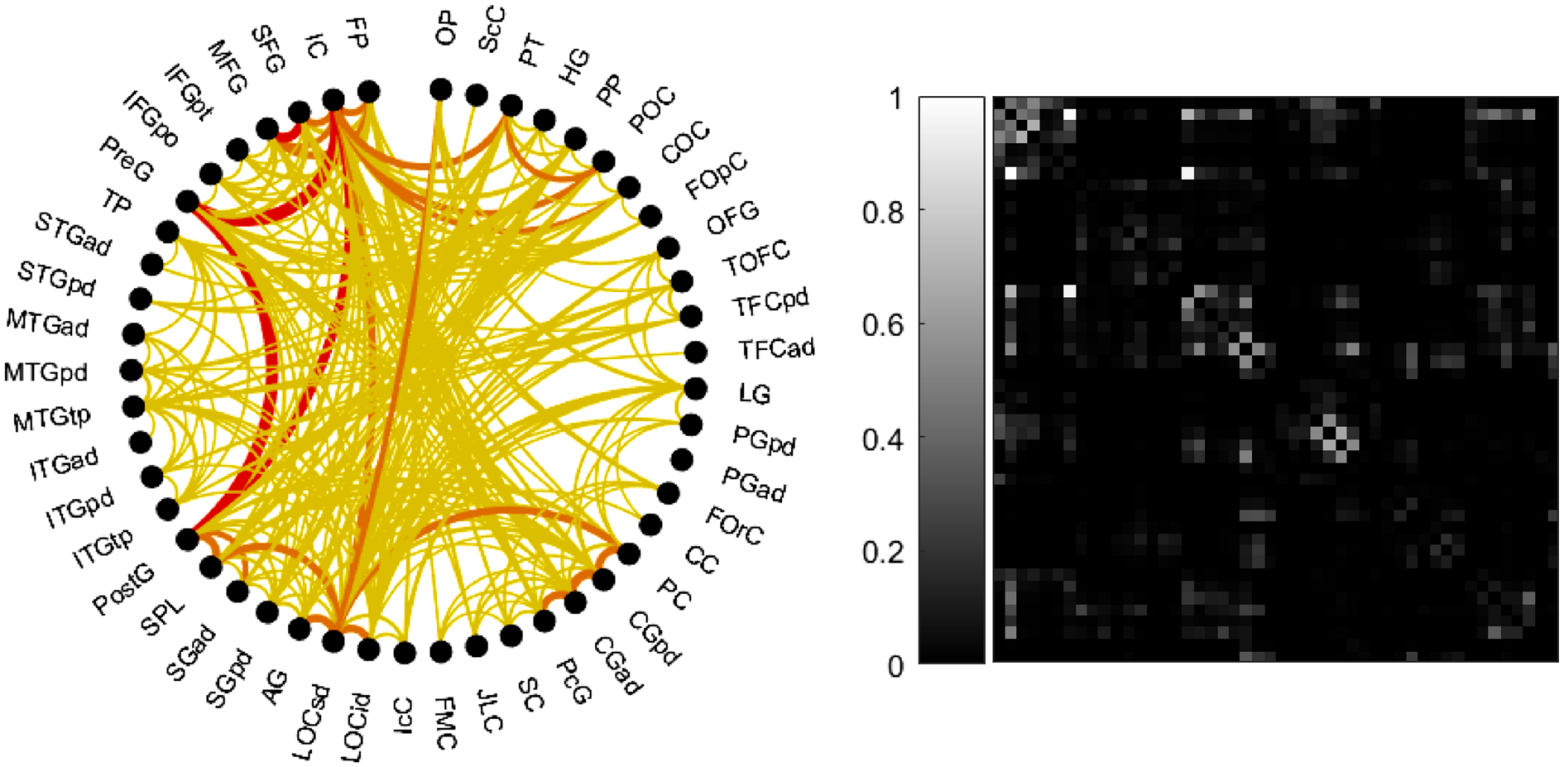
Connectogram (left) described by the structural connectivity matrix $A_0$ (right). For clarity, only 30 % of the edges (corresponding to the highest weights) are displayed. Edges are shown in yellow if the associated weight $a_{0ij} \in [0,0.33]$, in orange if $a_{0ij} \in [0.33,0.66]$, in red if $a_{0ij} \in [0.66,1]$. Nodes are labeled according to the HOA. Connectogram graphed with SPIDER-NET software^54^.

We consider a weighted and undirected network graph (described by the symmetric structural connectivity matrix $A_0$) with $N=48$ nodes (Fig. 4), each representing a cortical area according to the Harvard-Oxford Cortical Atlas (HOA); a list of the cortical areas is provided in “Supplemental Material”, Note 1). Edges represent long-range connections between the cortical areas.

#### Identification of clusters of nodes with coherent activity

For each correlation matrix *X*, we define the corresponding dissimilarity matrix as $D = \textbf{1}_N - X$ (where $\textbf{1}_N$ is an $N \times N$ matrix with all entries equal to 1). *D* is converted to vector form with Matlab’s *squareform* function and fed to Matlab’s *linkage* function with the *‘complete’* option (which implements the farthest neighbor method) in order to perform the hierarchical clustering. In this case, we identify $\mathcal {L}^*=\{\ell ^*_{13},\ell ^*_{18},\ell ^*_{21},\ell ^*_{31},\ell ^*_{39}\}$ (i.e., $k\in \{13,18,21,31,39\}$) as the set of local maxima of $\Psi _1(\ell )$, and maximum $\Psi _2$ for session $s^*=10$ (see Fig. 3). The level $\ell ^*_{13}$ ($\ell ^*_{39}$) corresponds to the coarsest (finest) scale of description of the brain areas’ correlations.

**Figure 5:**
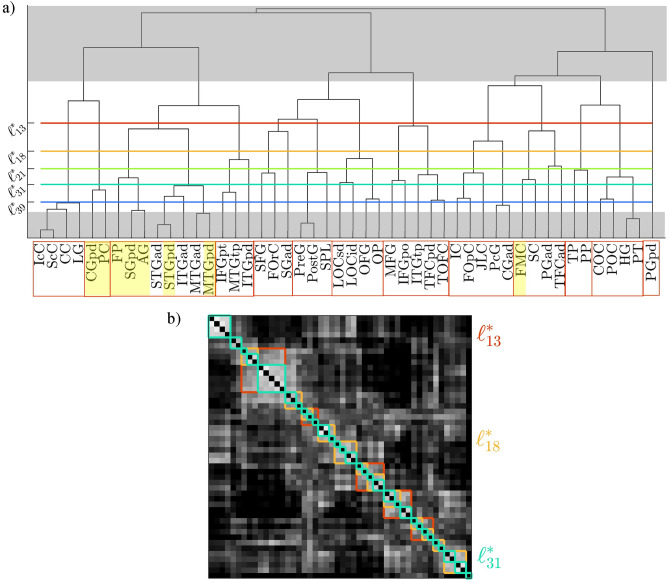
(**a**) Dendrogram associated with $X^*$. The levels $\ell ^*_k$ are marked by colored horizontal lines. Grey areas correspond to trivial partitions with too few or too many clusters. Nodes are labeled according to the HOA and their division in clusters corresponding to $\ell ^*_{13}$ is evidenced by red boxes. Nodes belonging to the DMN are highlighted in yellow. (**b**) Correlation matrix $X^*$ where the sets of clusters corresponding to levels $\ell ^*_{13}$ (red), $\ell ^*_{18}$ (orange), and $\ell ^*_{31}$ (teal) are highlighted.

Panel a of Fig. 5 shows the dendrogram associated with $X^*$, where the levels $\ell ^*_k$ are marked by colored horizontal lines. The sets of clusters corresponding to levels $\ell ^*_{13}$, $\ell ^*_{18}$, and $\ell ^*_{31}$ are highlighted over the correlation matrix $X^*$ in panel b. Nodes on the dendrogram are labeled according to the HOA and their division in clusters corresponding to $\ell ^*_{13}$ is evidenced by red boxes at the bottom of panel a. Nodes belonging to the default mode network (DMN), the first and most studied resting-state subnetwork^55–57^, are highlighted in yellow. We remark that we do not exploit neuroscientifically relevant a priori knowledge to identify the clusters, but only the functional connectivity matrices obtained from fMRI data. Nonetheless, we can observe that 5 out of the 8 cortical areas constituting the DMN, as listed in^58,59^, belong to the same cluster (the frontal pole, the angular gyrus, and the posterior divisions of the supramarginal gyrus, the middle temporal gyrus and the superior temporal gyrus), 2 constitute the adjacent cluster (the posterior division of the cingulate gyrus and the precuneous cortex), and only 1 (the frontal medial cortex) is isolated. This shows that we observe coherent behavior among the majority of the cortical areas belonging to the DMN.

Is the structural connectivity matrix $A_0$ compatible with the clustering observed in the functional connectivity matrix $X^*$? To verify this, we model the network nodes as Wilson-Cowan neural masses^44^ and the BOLD signal associated with each node by using the Balloon-Windkessel hemodynamic model of Friston and Harrison^60^. Details on the models and model parameters (set so that the nodes exhibit oscillatory behavior, in accordance with the local field potential gamma oscillations observed in cortical activity) can be found in Methods.

The initial conditions for all network simulations (performed with the *ode45* Matlab ODE solver) are set according to the following criteria: we want to give the same $n_{IC}$ initial conditions to the nodes belonging to the same target cluster, to enable the emergence of said cluster; at the same time, we want to perturb slightly these initial conditions to ensure that the state variables do not get stuck on an unstable orbit. For this reason, the initial conditions for the state variables of the nodes belonging to the same target cluster are defined as a common mean value, set randomly at each trial, plus Gaussian noise with a small standard deviation of $10^{-5}$. Initial conditions for the state variables of the BOLD model are simply set randomly at each trial, regardless of the target cluster configuration. The correlation matrices are calculated using Matlab’s *corrcoef* function, which returns an $N \times N$ matrix with correlation coefficients of pairs of variables as off-diagonal entries.

**Figure 6:**
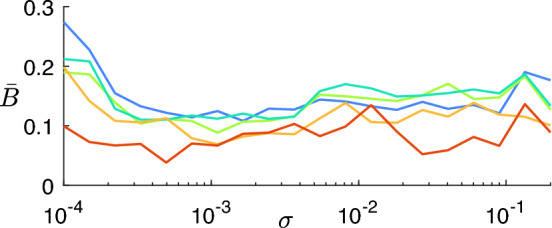
Comparison measure $\bar{B}$ between the cluster partition obtained from network simulations with synaptic weights $a_{0ij}$ (entries of $A_0$) and the target cluster partition associated with $X^*$, averaged over 30 random trials for different values of $\sigma$. Different colors refer to different levels $\ell _k^*$.

In our case study, the average comparison measure $\bar{B}$ is found to be low (always below 0.3) for all levels $\ell _k^*$ and for all values of $\sigma$, as shown in Fig. 6.

This highlights that using the information contained in the structural connectivity matrix $A_0$ ‘as is’ to model connection weights generates results that are incompatible with the observed functional connectivity. This finding is in agreement with other studies that have questioned the reliability of using connectivity matrices describing fiber density as a quantitative indication of connection weights^61–63^. Next, we show how the proposed method refines the structural connectivity matrix $A_0$ to make it compatible with the observed functional connectivity.

#### Refining the structural connectivity matrix according to synchronized clusters

**Figure 7:**
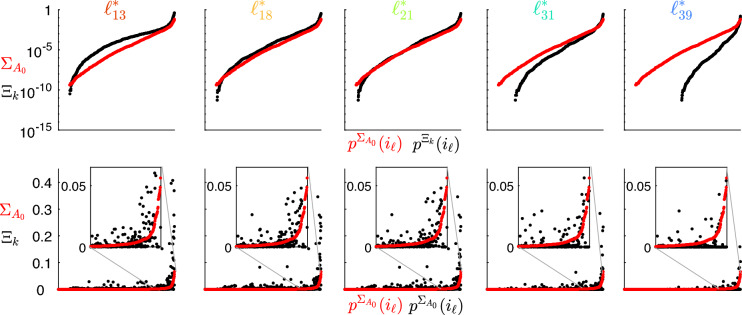
Entries of the matrices $\Sigma _{A_0}$ (red dots) and $\Xi _k$ (black dots), for all levels $\ell ^*_k$. Top panels: entries of both $\Sigma _{A_0}$ and $\Xi _k$ are displayed from the smallest to the largest and are plotted on a semi-logarithmic scale, with their linear indices $i_{\ell }$ ordered according to permutations $p^{\Sigma _{A_0}}$ and $p^{\Xi _k}$, respectively. Bottom panels: entries of $\Sigma _{A_0}$ are displayed on a linear scale from the smallest to the largest, with their linear indices $i_{\ell }$ ordered according to permutation $p^{\Sigma _{A_0}}$; entries of $\Xi _k$ are displayed following the same permutation.

We apply the proposed optimization procedure, thus obtaining the new structural connectivity matrices $A_k$. For the sake of comparison, we define the matrix $\Xi _k$ as the element-wise square difference between $A_k$ and $A_0$, i.e., $\Xi _{k_{ij}}=(a_{k_{ij}}-a_{0_{ij}})^2$. For matrices $\Sigma _{A_0}$ and $\Xi _k$ we introduce, respectively, the permutations $p^{\Sigma _{A_0}}$ and $p^{\Xi _k}$ of the linear index $i_{\ell }=i\cdot N+j$, which make the entries of the matrices ordered from the smallest to the largest. Fig. 7 (top panels) shows that the entries of the matrix $\Xi _k$ plotted against the permutated linear index $p^{\Xi _k}(i_{\ell })$ are comparable in magnitude to the entries of the matrix $\Sigma _{A_0}$ plotted against the permuted linear index $p^{\Sigma _{A_0}}(i_{\ell })$. We also note that the entries of the matrix $\Xi _k$ are smaller as the number of clusters increases because the optimization process changes less the original matrix $A_0$. This indicates that the optimized matrices $A_k$ do not differ from the original connectomes more than the uncertainty introduced by the measurement process. Bottom panels show that the square difference between the entries of the matrices $A_0$ and $A_k$ distributes similarly to the entries of the matrix $\Sigma _{A_0}$, i.e., the higher the uncertainty of a specific weight, the larger the change introduced by the optimization algorithm. In this case, linear indices of both matrices are ordered according to the permutation $p^{\Sigma _{A_0}}$.

#### Stability analysis through MSF and heterogeneity

**Figure 8:**
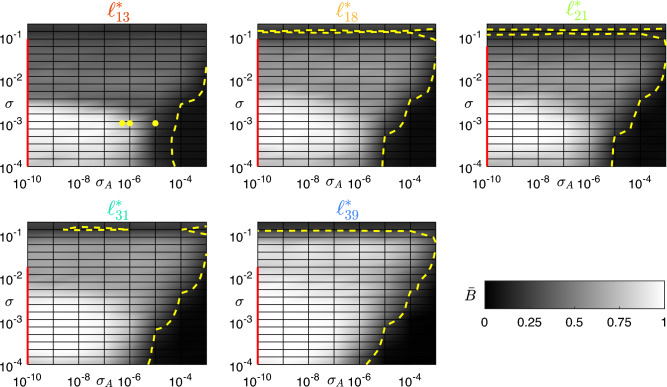
Comparison (through the average comparison measure $\bar{B}$) between the target cluster partition (derived from experimental data) and the partition obtained from network simulations with optimized matrices $A_k$ perturbed by Gaussian noise, for each level $\ell _k^*$; $\sigma _A$ on the abscissa denotes the noise standard deviation. Red bars on the vertical axis highlight the $\sigma$ (synaptic strength) for which the synchronous clusters are stable according to the MSF approach. Dashed yellow lines are the level curves delimiting the regions where $\bar{B}$ is higher than the maximum value obtained by simulating the network with the original structural connectivity matrix $A_0$ for the same level. Yellow dots mark the values of $\sigma$ and $\sigma _A$ used in Fig. 9.

**Figure 9:**
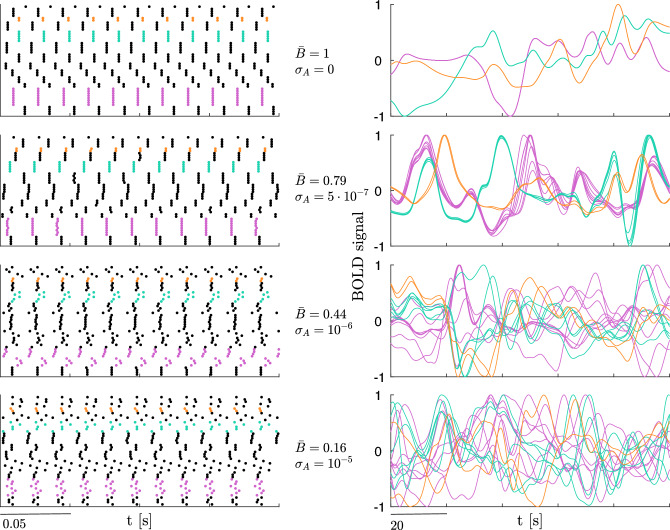
Raster plots (left) and normalized BOLD signals (right) for level $\ell ^*_{13}$, for different values of noise standard deviation $\sigma _A$ and for fixed $\sigma =10^{-3}$ (see yellow dots in Fig. 8). Points in the raster plots denote the peaks of excitatory subpopulation activity of each node, with nodes belonging to the same cluster represented as adjacent to each other. The normalized BOLD signals correspond to nodes in three sample clusters, chosen to showcase clusters of different sizes (pink: 8 nodes, teal: 5 nodes, orange: 2 nodes). Values of $\sigma _A$ increase from top to bottom and correspond to decreasing values of $\bar{B}$.

Figure 8 shows the results of the robustness analysis for all levels $\ell _k^*$, along with the intervals of $\sigma$ values for which the synchronous clusters for each level are stable according to the MSF approach (red lines). As expected, the average comparison measure $\bar{B}$ between the target partition (derived from experimental data) and the partition obtained by simulating the network with the optimized and perturbed structural connectivity matrices $A_k$ becomes lower as $\sigma _A$ grows. It can be observed that $\bar{B}$ is above the maximum value obtained by simulating the network with the original structural connectivity matrix $A_0$ in the large region enclosed within the yellow dashed curve. In this region, the optimized network (with connectivity matrix $A_k$) behaves in better accordance with the observed functional connectivity than the original network (with connectivity matrix $A_0$). Dark regions that fall outside the curve for low values of $\sigma _A$ are always beyond the stability interval identified through the MSF approach. This indicates that our results are robust to perturbations on the connection weights, also comparing the numerical values of $\sigma _A$ with the fact that the entries of $A_k$ have a mean of 0.029 and a standard deviation of 0.065, with minimum 0 and maximum 0.952. The raster plots and BOLD signals corresponding to specific values of $\sigma _A$ and $\bar{B}$ (yellow dots in Fig. 8) are shown in Fig. 9: the progressive desynchronization of clusters is apparent in both the raster plots and the BOLD signals as $\sigma _A$ increases and $\bar{B}$ decreases.

**Figure 10:**
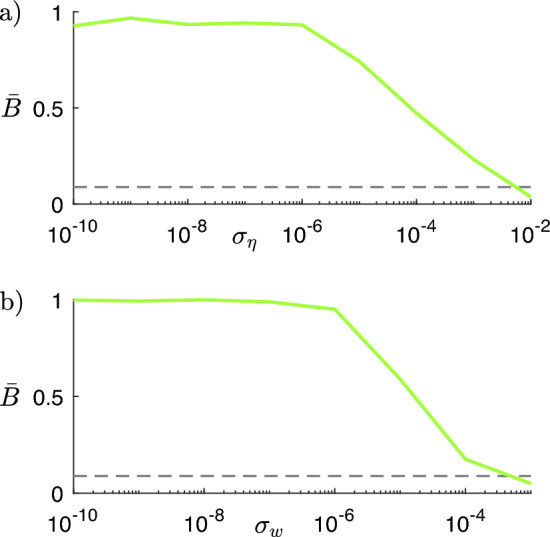
a) Comparison (through the average comparison measure $\bar{B}$) between the target cluster partition and the partition obtained from network simulations with connectivity matrix $A_{21}$ and a white Gaussian noise term $\eta _i(t)$ added to every node’s excitatory subpopulation input; the result is obtained for $\sigma =10^{-3}$. $\sigma _{\eta }$ is the noise signal standard deviation (see Eq. (7) in Methods). The gray dashed line marks the value of $\bar{B}$ obtained by simulating the network with the original structural connectivity matrix $A_0$ and without noise for the same level and same $\sigma$ value (see Fig. 6). b) Comparison (through the average comparison measure $\bar{B}$) between the target cluster partition and the partition obtained from network simulations with connectivity matrix $A_{21}$ and heterogeneous nodes. Heterogeneity is introduced by sampling the parameters $w_{EE}$, $w_{IE}$, $w_{EI}$ (see Eq. (7) in Methods) from Gaussian distributions with means $\mu _{w_{EE}}=3.5$, $\mu _{w_{IE}}=2.5$ and $\mu _{w_{EI}}=3.75$ and standard deviation $\sigma _w$. The result is obtained for $\sigma =10^{-3}$. The gray dashed line marks the value of $\bar{B}$ obtained by simulating the network with the original structural connectivity matrix $A_0$ and homogeneous nodes for the same level and same $\sigma$ value.

A similar analysis was carried out by adding a white Gaussian noise term $\eta _i(t)$ with standard deviation $\sigma _{\eta }$ to every node’s excitatory subpopulation input (see Eq. (7) in Methods). In this case, network simulations are performed with a fixed-step explicit Euler method, with integration step $dt=10^{-4}$. Panel a of Fig. 10 illustrates the results obtained for $\ell _{21}^*$ and strength $\sigma =10^{-3}$: similarly, $\bar{B}$ holds values close to 1 until $\sigma _{\eta }\le 10^{-6}$ . Note that $\bar{B}$ is higher than the value obtained by simulating the network with $A_0$ for the same level and the same $\sigma$ value, for standard deviation $\sigma _{\eta }$ up to about $10^{-2}$, which approaches the amplitude of the node input without noise; this means that the optimized structural connectivity matrix $A_k$ appears to be in better accordance with the observed functional connectivity than $A_0$, even for a relatively high noise level. This provides evidence of the robustness of the obtained results.

The robustness of the model was also tested by introducing heterogeneous node parameters. In particular, the model parameters that represent connection weights between inhibitory and excitatory subpopulations within each node (see Eq. (7) in Methods) were sampled from Gaussian distributions with standard deviation $\sigma _w$. Results are shown in panel b of Fig. 10: also in this scenario, the optimized structural connectivity matrix $A_k$ is in better accordance with the observed functional connectivity than $A_0$, up to about $\sigma _w=5\cdot 10^{-4}$.

#### Introducing delays

**Figure 11:**
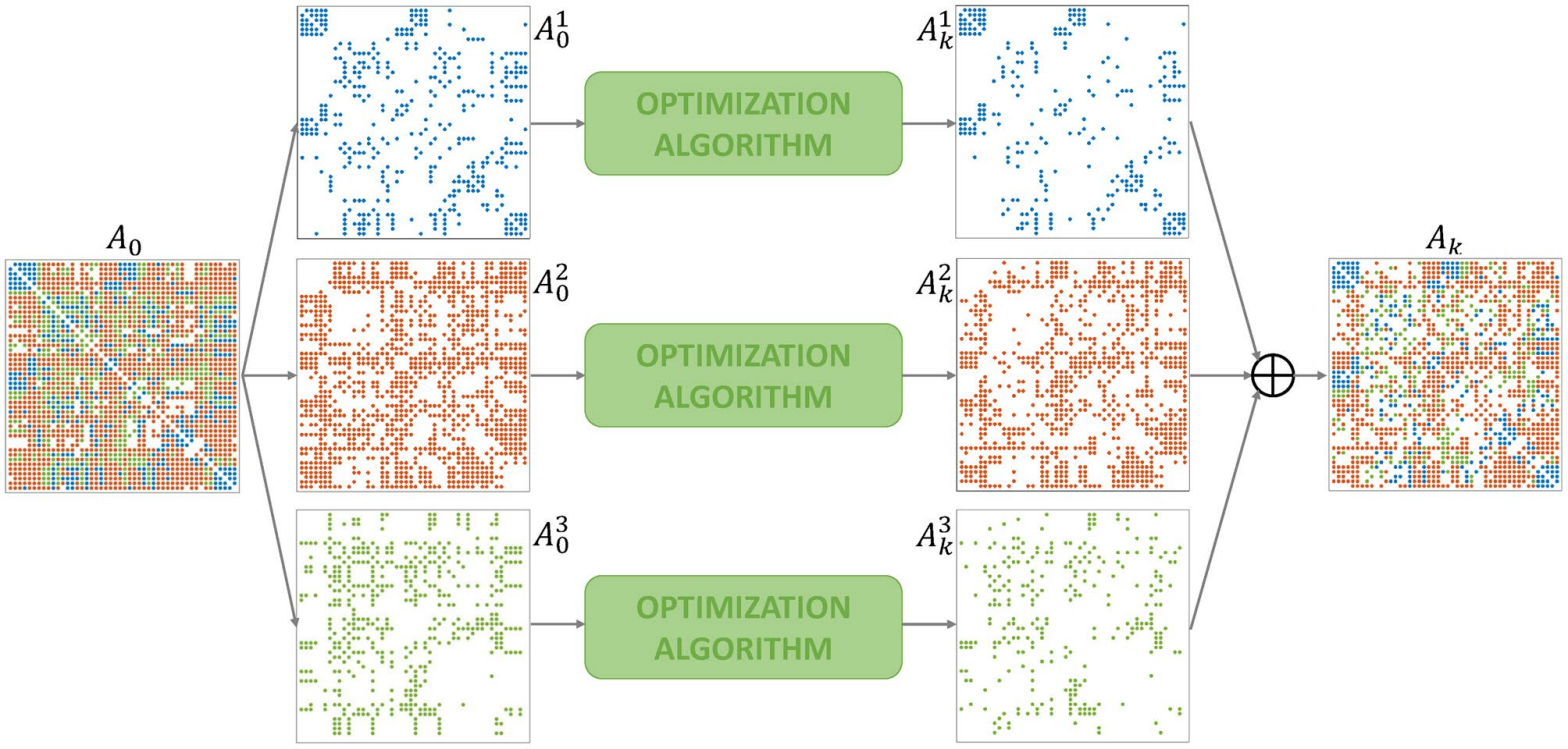
The structural connectivity matrix $A_0$ is split into 3 matrices $A_0^1$, $A_0^2$ and $A_0^3$, corresponding to link kinds with delay $\tau ^1=0.0204$ s, $\tau ^2=0.0518$ s and $\tau ^3=0.0832$ s, respectively. In this example, $k=21$.

The delays $\tau _{ij}$ are calculated by multiplying an $N\times N$ distance matrix by a propagation velocity of 1.5 m/s^64^; the distance matrix collects the pair-wise 3D euclidean distances between nodes, specifically between the centers of brain areas according to the HOA. Here, we quantize delays $\tau _{ij}$ over $L=3$ values and carry out the optimization on $A_0$ and the subsequent analysis for $\ell _{21}^*$. Our particular choice of $L=3$ aims to keep the complexity limited, while still obtaining good results, as shown in the following. The optimization process is summarized in Fig. 11: the structural connectivity matrix $A_0$ is split into $L=3$ matrices $A_0^l$, each corresponding to a coupling with delay $\tau ^l$. Each matrix $A_0^l$ is optimized individually, thus obtaining matrices $A_k^l$, which are then summed to obtain the final optimized structural connectivity matrix $A_k=\sum _l A_k^l$.

**Figure 12:**
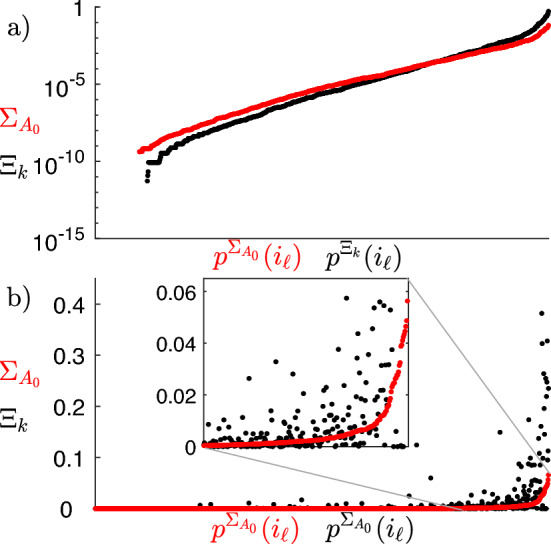
(**a**) Entries of the matrices $\Sigma _{A_0}$ (red dots) and $\Xi _k$ (black dots), for level $\ell ^*_{21}$. entries of both $\Sigma _{A_0}$ and $\Xi _k$ are displayed from the smallest to the largest and are plotted on a semi-logarithmic scale, with their linear indices $i_{\ell }$ ordered according to permutations $p^{\Sigma _{A_0}}$ and $p^{\Xi _k}$, respectively. (**b**) Entries of the matrices $\Sigma _{A_0}$ (red dots) and $\Xi _k$ (black dots), for level $\ell ^*_{21}$. entries of $\Sigma _{A_0}$ are displayed on a linear scale from the smallest to the largest, with their linear indices $i_{\ell }$ ordered according to permutation $p^{\Sigma _{A_0}}$; entries of $\Xi _k$ are displayed following the same permutation.

Panel a of Fig. 12 shows that the entries of the matrix $\Xi _k$ are still comparable with the entries of the matrix $\Sigma _{A_0}$, also considering $L=3$ kinds of connections. Moreover, panel b shows that the square difference between the entries of the matrices $A_0$ and $A_k$ distribute similarly to the entries of the matrix $\Sigma _{A_0}$.

**Figure 13:**
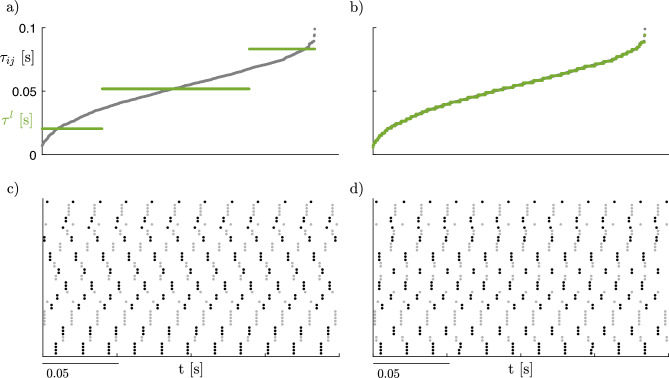
(**a**) Delays $\tau _{ij}$ sorted in ascending order (gray dots) and delays $\tau ^l$ quantized over 3 values (green lines). (**b**) Delays $\tau _{ij}$ sorted in ascending order (gray dots) and delays $\tau ^l$ quantized over 50 values (green lines). (**c**) Raster plot for level $\ell ^*_{21}$, for fixed $\sigma =10^{-3}$ and delays quantized over 3 values. Points in the raster plots denote the peaks of the excitatory subpopulation activity of each node, with nodes belonging to the same cluster represented as adjacent to each other; clusters are represented by alternating black and gray colors to aid visualization. d) Raster plot for level $\ell ^*_{21}$, for fixed $\sigma =10^{-3}$ and delays quantized over 50 values.

The stability analysis through the MSF verified that the cluster synchronous solution for level $\ell ^*_{21}$ is stable for $\sigma \in [10^{-4},0.1341]$, where $10^{-4}$ is the lowest value of $\sigma$ that we considered. The raster plot for $\sigma =10^{-3}$ and $L=3$, with delays quantized as in panel a of Fig. 13, is shown in panel c, evidencing the synchronization between the nodes in each cluster. Heterogeneity in delay values was reintroduced *a posteriori* to test the robustness of the result obtained for $L=3$. In particular, the delays were quantized over 50 values (panel b), which almost perfectly overlap with the original values of $\tau _{ij}$; we did not use the $\tau _{ij}$ values *as is*, because of the excessive computational burden. The corresponding raster plot is shown in panel d: as can be observed, heterogeneity in the delay values does not compromise the synchronization of the nodes within each cluster but influences the phase difference between clusters^65^. This analysis evidences that, from a theoretical standpoint, the proposed method can be generalized to account for heterogeneous delays in the network; however, the computational cost of simulating the network dynamics for fully heterogeneous delays becomes prohibitive, since the computational cost of the MSF approach increases linearly with *L*.

## Discussion

Neuroscientists monitor brain activity in several ways, using a variety of recording techniques in subjects under a plethora of health, neurophysiological and mental conditions. It becomes then important to integrate insights across diverse datasets to understand brain functions^66^. In this paper, we have proposed a method that reconciles dMRI and fMRI data of a given subject by using an approach based on nonlinear dynamics and cluster synchronization. As a proof of concept, we have applied the proposed method to dMRI and fMRI recordings of a healthy adult subject at resting state. We first found that the original structural connectivity matrix was not compatible with the observed synchronous clusters of brain areas. By modifying the connectivity weights by an amount that is comparable with the uncertainty introduced by the measuring process, we obtained a network model that is able to reproduce the observed synchronous clusters, also in the presence of heterogeneity in the network. The proposed method may pave the way toward a better interplay of complex network principles and neurophysiological data.

It has been shown that synchronous electrophysiological activity of groups of neural populations underlies essential motor or cognitive processes in normal brain function^67^. Moreover, abnormal synchronization of brain areas can be associated with a wide range of neurological and psychiatric disorders: in Parkinson’s disease, abnormal synchronization of neural circuits in the cortex and basal ganglia can be observed^68^, schizophrenia involves a disruption of neural synchrony^69^, and epileptic seizures are characterized by excessive and synchronized electrical activity in the brain^70^. Because of this, developing models that reproduce specific brain dynamics in terms of synchronized groups of brain areas, obtained by integrating structural and functional connectivity through cluster synchronization principles, is particularly relevant and could lead to a deeper understanding of brain functions.

While in the present paper we focused on a single hierarchical clustering for a given subject, we are aware that the study of dynamic functional connectivity reveals that brain activity switches between a set of states, where a finite number of clusterings are identifiable^24,71^. Future work will focus on generalizing the proposed method by modifying the structural connectivity optimization process so that the optimized matrix admits multiple observed clusterings. The stability analysis could then be carried out for each synchronous cluster solution, and simulations with different initial conditions could give information on the basin of attraction of each one, thus providing a model able to fully reproduce the observed dynamic functional connectivity.

Differently from other model-based approaches, such as dynamic causal modeling^72^ or Multivariate Ornstein-Uhlenbeck processes^73^, our approach does not aim at inferring effective connectivity, intended as a measure of the directional relationships in a dynamic model. In other words, we do not aim to generate a model able to retrace the spatiotemporal covariances of measured BOLD signals by inferring directed functional connections between nodes. Instead, we focus on reproducing the cluster synchronization observed among brain areas of a subject. The connections in our model are undirected and are heavily based on the measured structural connectivity: the small variations applied to the weights during the optimization process do not yield particular relevance from a neuroscientific point of view, and do not constitute a process of inference of unknown connections. These small variations, however, are enough to codify the balanced coloring condition in the structural connectivity matrix. This provides a network model that is strongly based on the physical connections and whose emergent dynamics exhibit the synchronous clusters observed in the functional connectivity of the subject. Notice that the proposed approach could nonetheless be generalized to asymmetric connectivity matrices detailing directed connections^30^.

Next we discuss in deeper detail some key points of the proposed approach to evidence its flexibility. In the presented case study, we used functional and structural connectivity matrices where network nodes are regions of interest (ROIs) defined according to the HOA. Our method can however be applied independently of the anatomical atlas used, or even exploit functional and structural connectivity matrices where nodes are spatial brain components estimated by independent components analysis (ICA)^74–76^, instead of ROIs. Moreover, functional connectivity matrices and the associated clustering could also be derived from other kinds of functional data (e.g. EEG, MEG)^77^.

Hierarchical clustering is an established method to identify clusters of nodes in brain networks^7,36–39^, due to the hierarchical modularity exhibited by the human brain^78^. However, the identification of the most appropriate number of clusters from a dendrogram is not straightforward. We carried out a multi-level approach and selected the levels of interest $\ell _k^*$ based on considerations derived from the experimental data. We remark that the proposed method remains valid regardless of the nature of the identified clusters, which could also be defined on the basis of neuroscientifically relevant *a priori* knowledge such as known resting state networks, like the DMN. In the absence of multiple scans for each subject or with no *a priori* knowledge, it is also possible to use other metrics to determine the optimal number of clusters, such as the silhouette score^79^ or the gap statistic^80^. In principle, one could also consider all levels, but this would greatly increase the computational cost of the method. Moreover, if multiple scans are not available, we cannot obtain information on the variance of the structural connectivity matrices among sessions. In this case, this information is simply not included in the optimization process: vector $\alpha$ in Eq. (2) is simply defined as the stacked columns of matrix $A_0$. In general, the cost function can be further customized, e.g., to impose that some elements of the connection matrix are not changed or to weigh differently the uncertainty about the entries of $A_0$.

In the proposed example, nodal dynamics are described by the Wilson-Cowan NMM^44^, with couplings as in^5,81^ and parameters as in^81^. In accordance with these and other studies^82,83^, we only modeled long-range excitatory projections targeting excitatory sub-populations, since we do not have information regarding whether and which connections directly target inhibitory subpopulations. The direct contribution of the synaptic current to the inhibitory subpopulation could be taken into account by adding the term $E_i^{syn}$ to the exponent of the second equation in Eq. system (7). Such modification of the Wilson-Cowan neural mass model would not alter the method proposed in the paper. Alternative models could also be employed, such as next-generation neural mass models, which exactly reproduce the macroscopic dynamics of heterogeneous spiking neural networks^84–88^. This would allow the use of heterogeneous parameters identified with high precision. The MSF step can be taken provided that the considered neural mass model falls under the proposed formalism or the formalism is appropriately generalized. The node homogeneity hypothesis needed to carry out the optimization of the structural connectivity matrix and the stability analysis would require the use of average parameters, under the assumption that parameters of distinct brain areas are not too different^89^. The proposed results have been obtained with data measured on healthy subjects in resting state. For non-healthy subjects, specific neural mass models should be used (e.g., the epileptor^90^ for epilepsy, or the model proposed in^91^ for Parkinson’s disease), but the proposed approach remains valid, *mutatis mutandis*. For healthy subjects exposed to a specific stimulus, an external input representing said stimulus could be added to the model. This would allow separating stimulus responses from ongoing activity^92^.

## Methods

### Definition of partition and coloring

Given a graph with *N* nodes, a partition $P=\{\mathcal {C}_1,\mathcal {C}_2,...,\mathcal {C}_k\}$ of the graph is defined as a subdivision of its node set into *k* clusters, with cluster $\mathcal {C}_p$ composed of $n_p$ nodes, $\sum _{p=1}^k n_p=N$ such that (i) there are no empty clusters, (ii) the clusters comprise all nodes and (iii) the clusters are pairwise disjoint. Each cluster can be identified through the labels of its constituting nodes and a given color. A network graph can admit many different colorings, each one describing a different partition.

### Hierarchical clustering and similarity indices $\Psi _1$ and $\Psi _2$

Depending on the level $\ell \in \{1, \dots , N\}$ at which the dendrogram is cut horizontally, the corresponding clustering is described by the sub-trees originating from each branch cut by the horizontal line: for example, in Fig. 2 the red line cuts the dendrogram at the level corresponding to 13 clusters. For each level $\ell$ of the dendrograms obtained from the hierarchical clustering of the correlation matrices, we calculate a cumulative (across sessions) similarity index $\Psi _1$:

5 $$\begin{aligned} \Psi _1(\ell )=\frac{1}{N_s}\sum _{i=1}^{N_s} B_i(\ell ) \end{aligned}$$

where $B_i(\ell )$ is the comparison measure between a single pair of functional connectivity matrices (corresponding to different fMRI sessions on the same subject) and $N_s$ is the total number of possible pairwise comparisons between the sessions; $B_i(\ell )=1$ when two partitions are identical and $B_i(\ell )=0$ when no pair of objects that appears in the same cluster in one partition is assigned to the same cluster in the other partition. We thus obtain *N* samples $\Psi _1(\ell )$. The clusters corresponding to each level $\ell ^*_k$ are established by computing a second cumulative (across the selected levels) similarity index $\Psi _2$ for each session:

6 $$\begin{aligned} \Psi _2(s)=\frac{1}{M_\ell }\sum _{\mathcal {L}^*} \frac{1}{M_f}\sum _{\begin{array}{c} i=1 \end{array}}^{M_f}B_i(s) \end{aligned}$$

where $B_i(s)$ is the comparison measure between the partition in the current session *s* and the partition in session *i*.

### Simulation procedure

1. We simulate the network dynamics and the corresponding BOLD signals for an array of values of $\sigma$ (see Eq. (1)), and repeat the procedure $n_{CI}$ times starting from different initial conditions chosen randomly.
2. For each simulation, we calculate the correlation matrix of the BOLD signals, discarding transient data. The resulting correlation matrix can be compared with the functional connectivity matrix $X^*$.
3. We apply the same hierarchical clustering that was applied on $X^*$ on each of the calculated correlation matrices, thus obtaining $M_\ell$ clusterings with *k* clusters, each corresponding to the level $\ell ^*_k$. We compare each obtained partition with the target partition associated with $X^*$, and compute the Fowlkes and Mallows matching index averaged over the $n_{CI}$ trials, which we denote as $\bar{B}$.

### Wilson–Cowan neural mass model

The dynamics of the *i*-th node is described by the equations:

7 $$\begin{aligned} {\left\{ \begin{array}{ll} \tau _E\dot{E}_i=-E_i+\frac{1}{1+e^{-c(w_{EE}E_i-w_{IE}I_i+P+\eta _i(t)+E^{syn}_i-\theta )}},\\ \tau _I\dot{I}_i=-I_i+\frac{1}{1+e^{-c(w_{EI}E_i-\theta )}}, \end{array}\right. } \end{aligned}$$

with

8 $$\begin{aligned} E^{syn}_i(t)=\sigma \sum _j a_{ij}E_j(t-\tau _{ij}), \end{aligned}$$

where the state variables $E_i(t)$ and $I_i(t)$ are the fraction of excitatory and inhibitory neurons firing per unit time at instant *t*, respectively; the weights $w_{uv}$, with $u,v=\{E,I\}$, describe the intra-population strength of connection from neuron type *u* to *v*; *c* and $\theta$ are, respectively, the gain and the threshold of the sigmoid; *P* is a spontaneous excitatory background input; $\eta _i(t)$ is a white Gaussian noise signal, kept null unless otherwise stated; $\tau _E,\tau _I$ control the timescales of the first-order kinetics; $E^{syn}_i$ is the synaptic input to node *i*, which is determined by the structural connectivity matrix entries $a_{ij}$ and the fraction of active excitatory cells in each *j*-th pre-synaptic population. Eq. (8) follows from the fact that long-range connections between cortical areas are only excitatory^93^. Delays $\tau _{ij}$ are assigned to each connection between nodes *i* and *j*, i.e., to each entry of the structural connectivity matrix $A_0$. We set the parameters as in^81^: $w_{EE}=3.5$, $w_{IE}=2.5$, $w_{EI}=3.75$, $c=4$, $\theta =1$, $P=0.34$. *P* is chosen so that the isolated node converges to an equilibrium point corresponding to a low activity state, but coupled nodes converge to limit cycles even for small coupling weights. A bifurcation diagram of the isolated node with respect to *P* is provided in the “Supplemental Material”, Note 3. Time constants are set to $\tau _E=$0.002 s and $\tau _I=$0.004 s, so that their ratio is as in^81^, but the nodes exhibit oscillations in the $\gamma$ range (around 40 Hz) as in^5^.

For a fixed value of *P*, the convergence of the steady-state trajectory to a limit cycle or to an equilibrium point depends on the value of the synaptic input $E^{syn}_i$, which in turn is influenced by the overall strength of the connections $\sigma$ in Eq. 8. In this paper, we consider the interval $\sigma \in [0.0001,0.2]$, which yields oscillatory behavior of the nodes. For $\sigma >0.2$ a growing portion of nodes ‘saturates’, converging to an equilibrium point, thus yielding constant $E_i(t)$ and $I_i(t)$ that are incompatible with the oscillating nature of the local field potential.

### BOLD model

We calculate the BOLD signal for each node *i* by using the Balloon-Windkessel hemodynamic model of Friston and Harrison^60^, which relates neural activity to perfusion changes. The model is described by the equations:

9 $$\begin{aligned} {\left\{ \begin{array}{ll} \dot{s}_i=z_i -\kappa s_i -\gamma (f_i-1),\\ \dot{f}_i=s_i,\\ \tau \dot{\nu }_i=f_i-\nu _i^{\frac{1}{\alpha }},\\ \tau \dot{q}_i=f_i\frac{1-(1-\rho )^{\frac{1}{f_i}}}{\rho }-\nu _i^{\frac{1}{\alpha }}\frac{q_i}{\nu _i}, \end{array}\right. } \end{aligned}$$

where $s_i$ is the vasodilatory signal, which increases according to the neuronal activity $z_i$ of the *i*-th region (in our case $z_i(t)=E_i(t)+I_i(t)$), and is subject to autoregulatory feedback; $f_i$ is the inflow, $\nu _i$ is the blood volume and $q_i$ is the deoxyhemoglobin content. Parameter $\alpha =0.32$ is the Grubb’s exponent^94^, $\rho =0.34$ is the resting oxygen extraction fraction and the other biophysical parameters are set to $\kappa =0.65$ per s, $\gamma =0.41$ per s and $\tau =0.98$ s, as per the mean values reported in^60^.

The BOLD signal is taken to be a static nonlinear function of $\nu _i$ and $q_i$:

10 $$\begin{aligned} y_i=V_0(7\rho (1-q_i)+2(1-\frac{q_i}{\nu _i})+(2\rho _i-0.2)(1-\nu _i)) \end{aligned}$$

where $V_0=0.02$ is the resting blood volume fraction^60^.

### Master stability function

The cluster synchronization state obeys the following equation:

11 $$\begin{aligned} \dot{\pmb {s}}_p(t)= \pmb {F}(\pmb {s}_p(t))+\pmb {\Gamma }\Bigg (\pmb {s}_p(t),\sigma \sum _l\sum _{q} r_{pq}^l\pmb {G}(\pmb {s}_q(t-\tau ^l))\Bigg ), \end{aligned}$$

where the $k-$dimensional matrix $R^l=\{r_{pq}^l\}$ is the quotient matrix, such that $r_{pq}^l=\sum _{j \in \mathcal {C}_q} a_{ij}^l$ ($i \in \mathcal {C}_p, \quad p,q=1,2,\ldots , k$). We preliminarily define

12 $$\begin{aligned} \begin{array}{c} D\tilde{\pmb {F}}(\pmb {s}_p(t)) =D\pmb {F}(\pmb {s}_p(t)) + D\pmb {\Gamma }_1 \Bigg (\pmb {s}_p(t), \sigma \displaystyle {\sum _l\sum _{q}} r_{pq}^l\pmb {G}(\pmb {s}_q(t-\tau ^l))\Bigg ) \end{array} \end{aligned}$$

and

13 $$\begin{aligned} \begin{array}{c} D\tilde{\pmb {\Gamma }}_p = D\pmb {\Gamma }_2\Bigg (\pmb {s}_p(t),\sigma \displaystyle {\sum _l\sum _q} r_{pq}^l\pmb {G}(\pmb {s}_q(t-\tau ^l))\Bigg ), \end{array} \end{aligned}$$

where $D\pmb {F}$ is the $m \times m$ Jacobian of the nodes’ vector field and the *m*-dimensional matrix $D\pmb {\Gamma }_1$ ($D\pmb {\Gamma }_2$) is the derivative of $\pmb {\Gamma }$ with respect to its first (second) argument. The linearized equations governing the dynamics of the perturbations about the synchronous solution $\pmb {s}_p(t)$ can be written as:

14 $$\begin{aligned} \begin{array}{cc} \dot{\pmb {w}}_{i}(t) = D\tilde{\pmb {F}}(\pmb {s}_p(t)) \pmb {w}_{i}(t)+ D\tilde{\pmb {\Gamma }}_p \sigma \displaystyle {\sum _l} \left[ \displaystyle {\sum _{q=1}^{k}} D\pmb {G}(\pmb {s}_q(t-\tau ^l)) \displaystyle {\sum _{j \in \mathcal {C}_q}} a_{ij}^l\pmb {w}_j(t-\tau ^l) \right] \end{array} \end{aligned}$$

Now we can rewrite Eqs. (14) in vector form by stacking all the state perturbation vectors together in one vector ${\pmb {W}(t)}$. Moreover, we introduce the $N \times N$ diagonal matrix $E_p$, which is the cluster indicator matrix: $E_p$ has entries $E_{p,ii}=1$, if node $i \in \mathcal {C}_p$, 0 otherwise, i.e., this matrix identifies all the nodes *i*’s that belong to cluster $\mathcal {C}_p$. Therefore, we have:

15 $$\begin{aligned} \begin{array}{cc} \dot{\pmb {W}} (t)= \left[ \displaystyle {\sum _{p=1}^k} E_p \otimes D{\tilde{\pmb {F}}}(\pmb {s}_p(t)) \right] \pmb {W}(t) \\ \\ + \left( \displaystyle {\sum _{p=1}^k} E_p \otimes D{\tilde{\pmb {\Gamma }}}_p\right) \sigma \displaystyle {\sum _l} \left[ \left( A^l\otimes I_n \sum _{q=1}^{k} E_q \otimes \pmb {G}(\pmb {s}_q(t-\tau ^l)) \right) \pmb {W}(t-\tau ^l)\right] \end{array} \end{aligned}$$

We compute the canonical transformation matrix *T* as the orthogonal matrix that simultaneously block-diagonalizes the matrices $A^1$, $A^2$,$\ldots$, $A^L$, $E_1$, $E_2$,$\ldots$, $E_C$ into *D* diagonal blocks, $$T=\left( \begin{array}{c} T_{\parallel } \\ T_{\bot } \\ \end{array} \right) = \mathcal {SBD}(A^1,A^2,\ldots ,A^L,E_1,E_2,\ldots ,E_C)$$. Application of the matrix *T* yields $TA^lT^{-1}=\hat{A}^l$, where $\hat{A}^l=\hat{A}^l_\parallel \oplus \hat{A}^l_\bot = \oplus _{j=1}^{D} \hat{A}^l_j$. The symbol $\oplus$ denotes the direct sum of matrices and the blocks $\hat{A}^l_j$ $j=1,2,\ldots , D$ have the same dimension. We remark that $\hat{A}^l_\parallel =\hat{A}^l_1$ and $\hat{A}^l_\bot = \oplus _{j=2}^{D} \hat{A^l}_j$. Moreover, we have that $T E_p T^{-1}=E_p$.

By using matrix *T*, for the transverse perturbations we obtain,

16 $$\begin{aligned} \begin{array}{c} \dot{\pmb \eta }_\bot (t)= \underbrace{\left[ \displaystyle {\sum _{p=1}^k} E_{p\bot } \otimes D{\tilde{\pmb {F}}}(\pmb {s}_p(t)) \right] }_{\displaystyle {\rho _1(\left\{ \pmb {s}_p(t) \right\} )}} \pmb \eta _\bot (t)\\+ \displaystyle {\sum _l} \bigg [ \underbrace{ \left( \displaystyle {\sum _{p=1}^k} E_{p\bot } \otimes D{\tilde{\pmb {\Gamma }}}_p \right) \sigma \left( \hat{A}^l_{\bot } \otimes I_n \sum _{q=1}^{k} E_{q\bot } \otimes D\pmb {G}(\pmb {s}_q(t-\tau ^l)) \right) }_{\displaystyle {\rho _2^l(\left\{ \pmb {s}_p(t) \right\} )}} \pmb {\eta }_\bot (t-\tau ^l) \bigg ] \end{array} \end{aligned}$$

where the block-diagonal matrix marked with $\bot$ is a minor of the complete matrix $\hat{A}^l= TA^lT^{-1}$, containing only the blocks related to the transverse perturbations.

### Supplementary Information

Supplementary Information.

## Data Availability

The datasets analyzed during the current study are available in the “M2G: Reliable Human Connectomes At Scale” repository, https://neurodata.io/mri/.
