# Supplementary material for: Bridging functional and anatomical neural connectivity through cluster synchronization
Source: Sci Rep. 2023 Dec 17;13:22430. doi: 10.1038/s41598-023-49746-2 (PMC10725511; doi:10.1038/s41598-023-49746-2)
Supplement: Supplementary file 1 — Supplementary Information. [file 41598_2023_49746_MOESM1_ESM.pdf]

Supplemental Material of “Bridging functional and anatomical neural connectivity through cluster synchronization”

Valentina Baruzzi[1], Matteo Lodi[1], Francesco Sorrentino[2], and Marco Storace[1,*]

[1]DITEN, University of Genoa, Via Opera Pia 11a, I-16145, Genova, Italy
[2]Mechanical Engineering Department, University of New Mexico, Albuquerque, NM 87131, USA
[*]marco.storace@unige.it

Note 1

This note contains Fig. S1, Showing the numbering of cortical areas according to the Harvard-Oxford Cortical Structural Atlas (RRID:SCR_001476).

1	FP	Frontal Pole	25	FMC	Frontal Medial Cortex
2	IC	Insular Cortex	26	JLC	Justapositional Lobule Cortex
3	SFG	Superior Frontal Gyrus	27	SC	Subcallosal Cortex
4	MFG	Middle Frontal Gyrus	28	PcG	Paracingulate Gyrus
5	IFGpt	Inferior Frontal Gyrus pars triangularis	29	CGad	Cingulate Gyrus anterior division
6	IFGpo	Inferior Frontal Gyrus pars opercularis	30	CGpd	Cingulate Gyrus posterior division
7	PreG	Precentral Gyrus	31	PC	Precuneous Cortex
8	TP	Temporal Pole	32	CC	Cuneal Cortex
9	STGad	Superior Temporal Gyrus anterior division	33	FOrC	Frontal Orbital Cortex
10	STGpd	Superior Temporal Gyrus posterior division	34	PGad	Parahippocampal Gyrus anterior division
11	MTGad	Middle Temporal Gyrus anterior division	35	PGpd	Parahippocampal Gyrus posterior division
12	MTGpd	Middle Temporal Gyrus posterior division	36	LG	Lingual Gyrus
13	MTGtp	Middle Temporal Gyrus temporooccipital part	37	TFCad	Temporal Fusiform Cortex anterior division
14	ITGad	Inferior Temporal Gyrus anterior division	38	TFCpd	Temporal Fusiform Cortex posterior division
15	ITGpd	Inferior Temporal Gyrus posterior division	39	TOFC	Temporal Occipital Fusiform Cortex
16	ITGtp	Inferior Temporal Gyrus temporooccipital part	40	OFG	Occipital Fusiform Gyrus
17	PostG	Postcentral Gyrus	41	FOpC	Frontal Operculum Cortex
18	SPL	Superior Parietal Lobule	42	COC	Central Opercular Cortex
19	SGad	Supramarginal Gyrus anterior division	43	POC	Parietal Operculum Cortex
20	SGpd	Supramarginal Gyrus posterior division	44	PP	Planum Polare
21	AG	Angular Gyrus	45	HG	Heschl's Gyrus (includes H1 and H2)
22	LOCsd	Lateral Occipital Cortex superior division	46	PT	Planum Temporale
23	LOCid	Lateral Occipital Cortex inferior division	47	ScC	Supracalcarine Cortex
24	IcC	Intracalcaring Cortex	48	OP	Occipital Pole

Figure S1. Numbering of cortical areas according to the Harvard-Oxford Cortical Structural Atlas (RRID:SCR_001476).

Note 2

This note contains the results of the application of the proposed method to functional and structural data of two additional subjects of the HNU1 dataset[1] in the Neurodata MRI Cloud database[2].

The left side of Fig. S2 shows the $\Psi_1(\ell)$ index for all subjects of the dataset, color-coded according to the bottom colorbar. The index peaks, which identify the sets of selected levels $\mathscr{L}^*$, are marked with red dots. In the main paper, we present results for subject 0025452, which showed the highest similarity index Ψ_1 averaged across levels. Here, we present equivalent results for subjects 0025428 and 0025447, which show the second and third highest average similarity index Ψ_1, respectively. This criterion of selection favors subjects that exhibit good agreement among clusterizations obtained from different fMRI sessions. In other words, we considered subjects that exhibited a similar resting state activity, in terms of groups of synchronized brain areas, across different recordings. The right side of Fig. S2 displays the isolated $\Psi_1(\ell)$ curves of the two selected subjects. According to the $\Psi_2(s)$ curves calculated for these subjects, we selected the X^* correlation matrices as follows: the one associated with session 1 for subject 0025428, and the one associated with session 5 for subject 0025447.

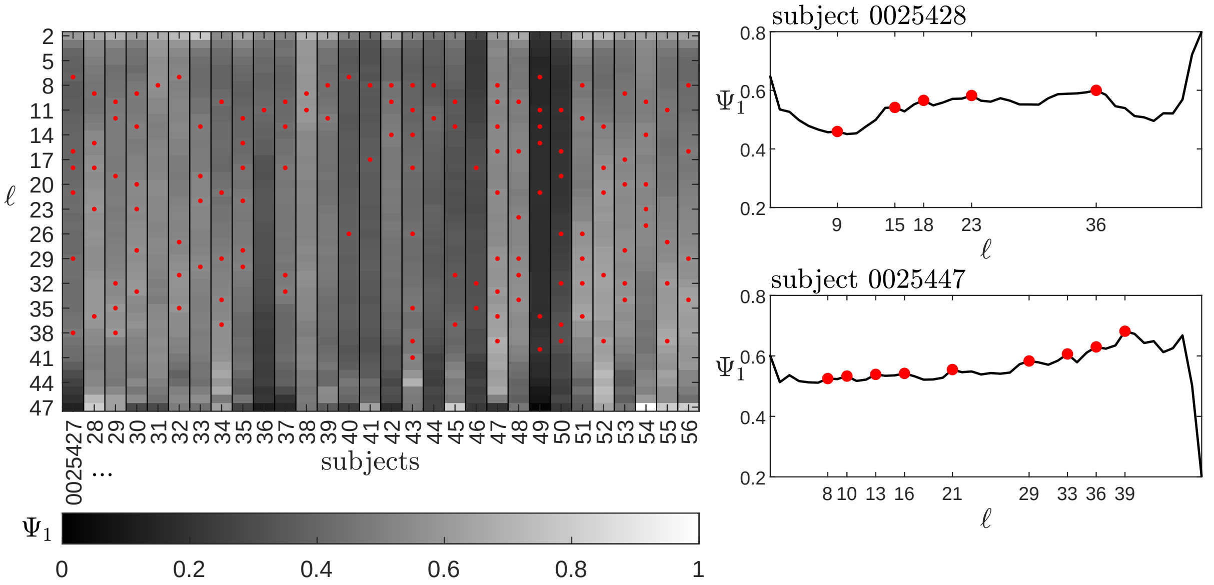

Figure S2. Left panel: $\Psi_1(\ell)$ index for all subjects of the HNU1 dataset[1], color-coded according to the bottom colorbar. Right panels: $\Psi_1(\ell)$ curves for subjects 0025428 and 0025447, which show the second and third highest average similarity index Ψ_1, respectively. Red dots: $\Psi_1(\ell)$ peaks, which identify the sets of selected levels $\mathscr{L}^*$.

Fig. S3 shows the dendrograms obtained from the hierarchical clustering applied on matrices X^* for both subjects, where the central levels of the sets $\mathscr{L}^*$ (level ℓ^*_{18} for subject 0025428 and level ℓ^*_{21} for subject 0025447) are marked by red horizontal lines. Nodes on the dendrogram are labeled according to the HOA.

We remark that matrix Ξ_k is defined as the element-wise square difference between A_k and A_0, i.e., $\Xi_{k_{ij}} = (a_{k_{ij}} - a_{0_{ij}})^2$ and that for matrices Σ_{A_0} and Ξ_k we introduce, respectively, the permutations $p^{\Sigma_{A_0}}$ and p^{Ξ_k} of the linear index $i_\ell = i \cdot N + j$, which make the entries of the matrices ordered from the smallest to the largest. Fig. S4 confirms that, for both subjects, the entries of the matrix Ξ_k are comparable with the entries of the matrix Σ_{A_0} when plotted against the permutated linear index $p^{\Xi_k}(i_\ell)$ and $p^{\Sigma_{A_0}}(i_\ell)$, respectively. Moreover, the square difference between the entries of the matrices A_0 and A_k distributes similarly to the entries of the matrix Σ_{A_0}, i.e., the higher the uncertainty of a specific weight, the larger the change introduced by the optimization algorithm. In this case, linear indices of both matrices are ordered according to the permutation $p^{\Sigma_{A_0}}$.

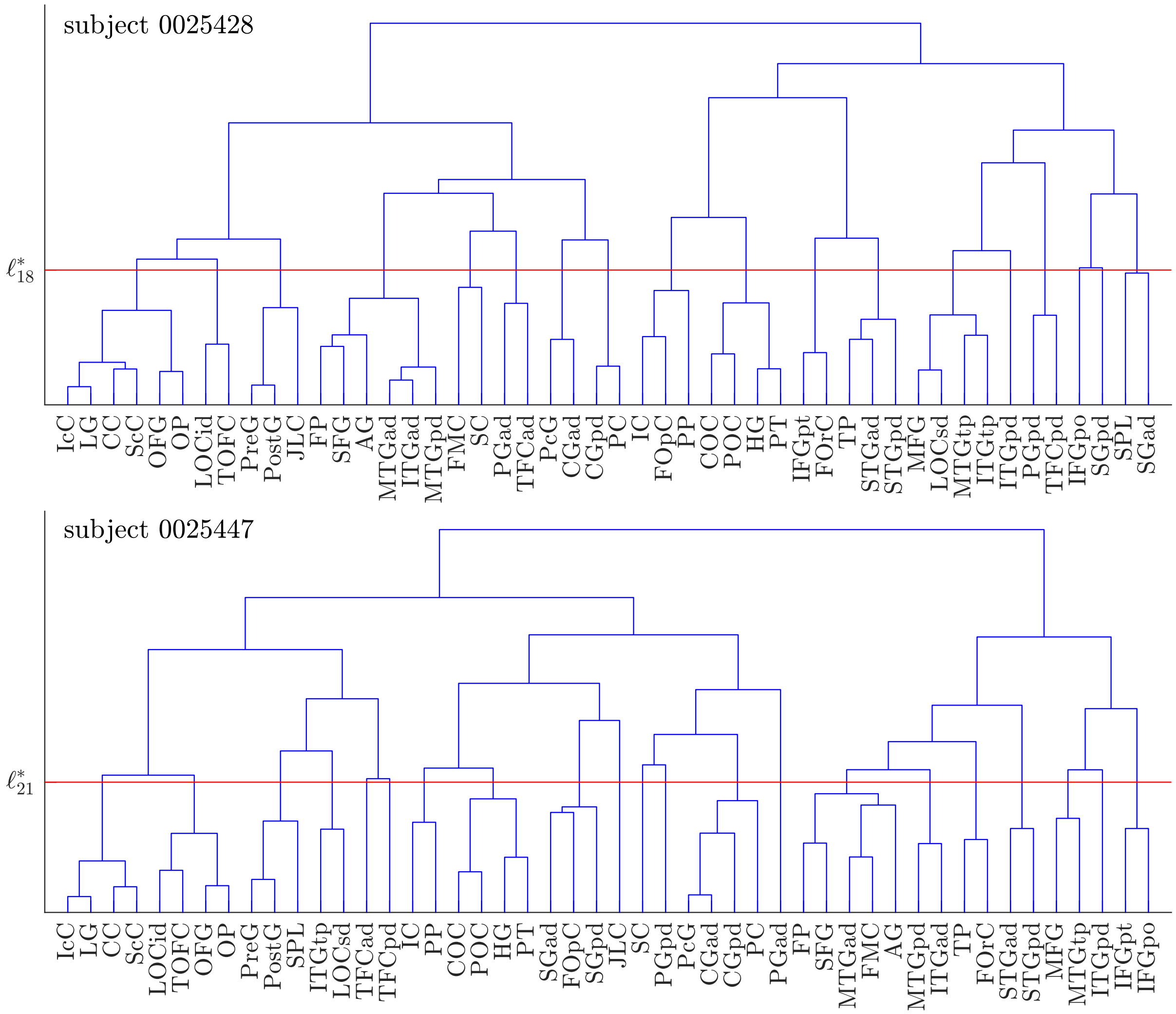

Figure S3. Dendrograms obtained from the hierarchical clustering applied on matrices X^* for subjects 0025428 and 0025447. Red horizontal lines: central levels of the sets $\mathscr{L}^*$ (level ℓ^*_{18} for subject 0025428 and level ℓ^*_{21} for subject 0025447). Nodes on the dendrogram are labeled according to the HOA.

Figure S5 shows the results of the robustness analysis for level ℓ^*_{18} of subject 0025428 and ℓ^*_{21} of subject 0025447. As expected, the average comparison measure $\bar{B}$ between the target partition (derived from experimental data) and the partition obtained by simulating the network with the optimized and perturbed structural connectivity matrices A_k becomes lower as σ_A grows. It can be observed that $\bar{B}$ is above the maximum value obtained by simulating the network with the original structural connectivity matrix A_0 in the large region enclosed within the yellow dashed curve. In this region, the optimized network (with connectivity matrix A_k) behaves in better accordance with the observed functional connectivity than the original network (with connectivity matrix A_0). This confirms that the model is robust to perturbations in the connection weights.

These results are aligned with the case study presented in the main paper, supporting the reliability of the proposed method.

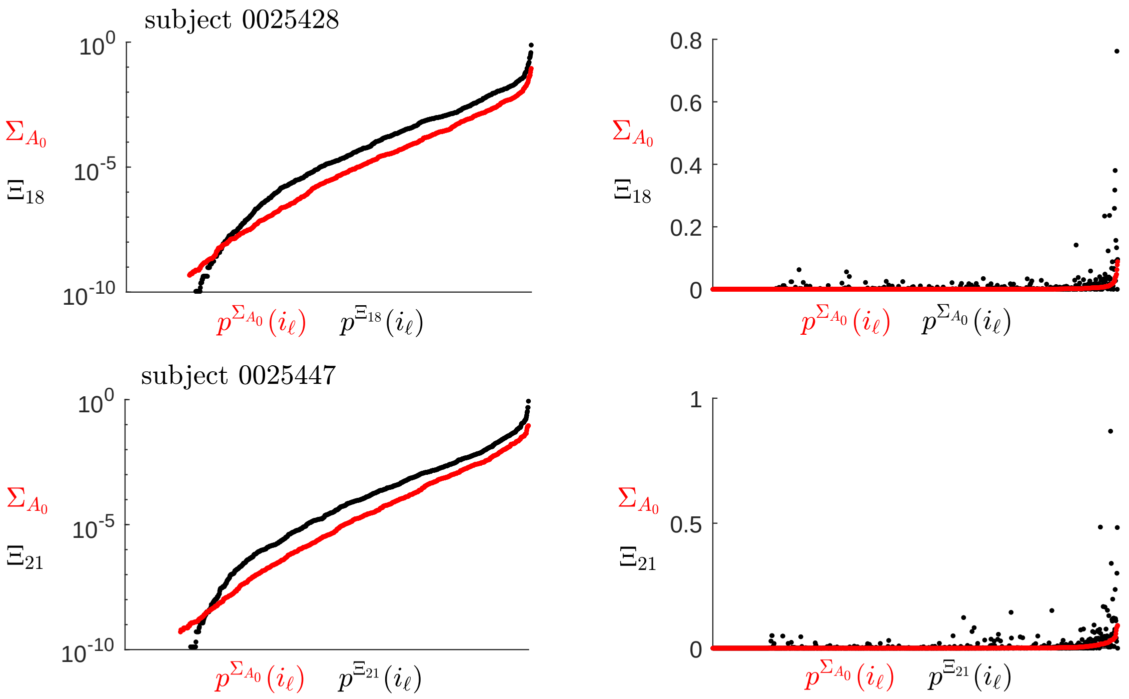

Figure S4. Entries of the matrices Σ_{A_0} (red dots) and Ξ_k (black dots) for subjects 0025428 (top panels) and 0025447 (bottom panels). Left panels: entries of both Σ_{A_0} and Ξ_k are displayed from the smallest to the largest and are plotted on a semi-logarithmic scale, with their linear indices i_ℓ ordered according to permutations $p^{\Sigma_{A_0}}$ and p^{Ξ_k}, respectively. Right panels: entries of Σ_{A_0} are displayed on a linear scale from the smallest to the largest, with their linear indices i_ℓ ordered according to permutation $p^{\Sigma_{A_0}}$; entries of Ξ_k are displayed following the same permutation.

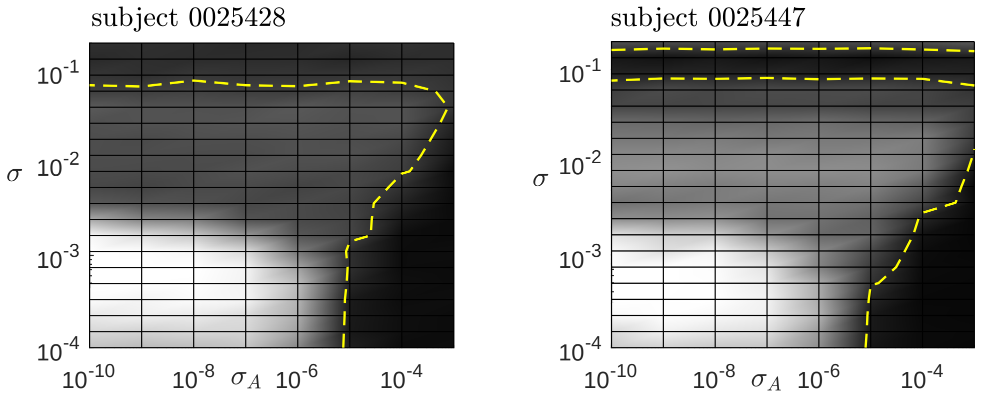

Figure S5. Robustness analysis for level ℓ^*_{18} of subject 0025428 and ℓ^*_{21} of subject 0025447. Comparison (through the average comparison measure $\bar{B}$) between the target cluster partition (derived from experimental data) and the partition obtained from network simulations with optimized matrices A_k perturbed by Gaussian noise, for the selected level ℓ^*_k; σ_A on the abscissa denotes the noise standard deviation. Red bars on the vertical axis highlight the σ (synaptic strength) for which the synchronous clusters are stable according to the MSF approach. Dashed yellow lines are the level curves delimiting the regions where $\bar{B}$ is higher than the maximum value obtained by simulating the network with the original structural connectivity matrix A_0 for the same level.

Note 3

This note contains Fig. S6, showing the bifurcation diagram of an isolated Wilson-Cowan oscillator with respect to P. The other parameters are set as follows: $w_{EE} = 3.5$, $w_{IE} = 2.5$, $w_{EI} = 3.75$, $c = 4$, $\theta = 1$, τ_E =0.002 s and τ_I =0.004 s. Stable attractors are represented with solid green lines, unstable attractors are represented with red dashed lines. Bifurcation points are evidenced with black stars. The bifurcation diagram was computed using the MatCont toolbox[3].

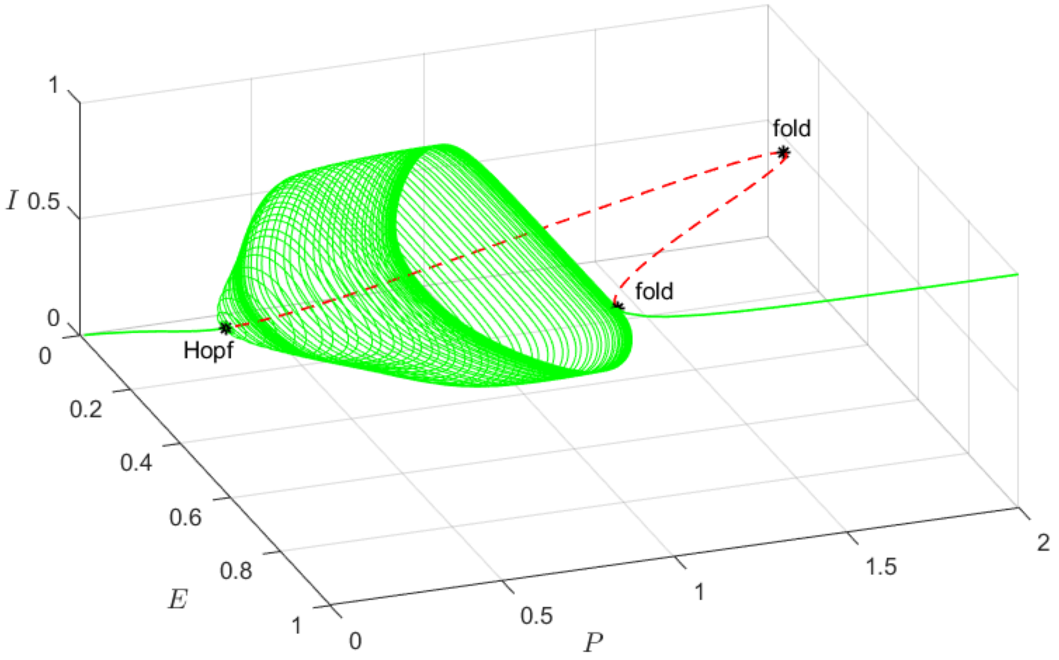

Figure S6. Bifurcation diagram of an isolated Wilson-Cowan oscillator with respect to parameter P. The other parameters are $w_{EE} = 3.5$, $w_{IE} = 2.5$, $w_{EI} = 3.75$, $c = 4$, $\theta = 1$, τ_E =0.002 s and τ_I =0.004 s. Solid green lines: Stable attractors. Red dashed lines: unstable attractors. Black stars: bifurcation points.

References

1. Zuo, X.-N. *et al.* An open science resource for establishing reliability and reproducibility in functional connectomics. *Sci. data* **1**, 1–13 (2014).
2. MRI Cloud. https://neurodata.io/mri/. Accessed: 21-07-2022.
3. Dhooge, A., Govaerts, W., Kuznetsov, Y. A., Meijer, H. G. E. & Sautois, B. New features of the software matcont for bifurcation analysis of dynamical systems. *Math. Comput. Model. Dyn. Syst.* **14**, 147–175 (2008).
